# Amplifying under-represented perspectives

**DOI:** 10.1038/s41467-022-29269-6

**Published:** 2022-03-31

## Abstract

Our Community Voices series aims to highlight the challenges faced by under-represented groups in scientific research and to provide a platform to share possible solutions, to work towards a more diverse and equitable research landscape.

We believe that a diverse and equitable research community is the foundation for discoveries that benefit the global community. For many, including ourselves, the last 2 years have highlighted, yet again, that much more needs to be done to accelerate and support the growth of a research community representative of the world population. This is necessary to ensure research questions and solutions integrate prior knowledge, are relevant to all and can help tackle biases built into the system. With this editorial, we launch our Community Voices series as a space for under-represented communities of researchers to call attention to the issues they face and bring forward potential solutions, but this is just the start.

Our publisher, Springer Nature, to which we belong as part of Nature Portfolio has been taking stock of our working practices. We have put into place an inclusive code of conduct that includes committing to inviting equal percentages of women and men as speakers and having no men-only panels at our events. In December 2020, we announced our commitment to increase diversity in our organization, and last autumn, we followed up with further initiatives including how we want to work with others in the industry by signing up the Joint Commitment for Action on Inclusion and Diversity in Publishing spearheaded by the Royal Society of Chemistry.

When *Nature Communications* editors reach out to reviewers with the expertise needed to evaluate the manuscripts we receive, we strive to keep diversity in mind. Since 2018, we have been asking our reviewers to share information about their gender, geographical location and career stage, on a voluntary basis, to inform our overall efforts to increase diversity in the review process as a whole. We have recently looked back at 4 years of this data, representing nearly 100,000 responses, which showed that we have improved in some areas but progress has been frustratingly slow. In 2018, 18% of our reviewers identified as women, a number which increased to 20% by 2021 (7% preferred not to share this information in both 2018 and 2021). We also know that in 2018, 57% were professors and 77% worked in countries in North America and Europe while in 2021, 42% were professors and 71% worked in North America and Europe.

The message is clear: we must try harder to ensure that a diverse representation of researchers is involved in our peer-review process. Why, despite our efforts, are we struggling to see the impact that we are seeking? We know that women were more affected in their research activities by the pandemic, as outlined in a recent Research Comment from Dashun Wang, and we understand that many researchers from under-represented groups are often over-committed and may not be able to take on additional duties such as reviewing manuscripts at this time.

We are open to extending the time for review when needed to accommodate the multiple demands. We would also encourage invited reviewers to work with an early career researcher (ECR) in their laboratory to review with them; you should let us know to whom you are offering this teaching opportunity so that we can recognize their work as co-reviewer. We have ourselves engaged with ECRs in the last 2 years with the view of supporting those who may not have had an opportunity to review manuscripts. Within our peer-review program, following a webinar aimed at providing guidance on how to review manuscripts, editors send selected ECRs a manuscript under review at the journal (which is considered through the normal processes of contacting independent experts) and the editors then provide personalized feedback to the ECRs on their report. The ECRs also gain access to the reports sent by the other reviewers, giving them a point of comparison. We were delighted to receive more than 1500 applicants from all over the world to our program last year and we will report soon on last year’s scheme and will announce further initiatives aimed at supporting ECRs.

We have also been reflecting on how we can help to amplify voices from under-represented communities, and provide a platform for researchers to propose possible solutions to further support diversity, equity and inclusion in research.

In 2021, we published a Perspective by Hendratta Ali et al. proposing an actionable anti-racism plan for geoscience organizations and we engaged with researchers on how to build safe spaces for the LGBTQ+ community in geoscience. We have been working with groups of researchers to examine what are in their view the key issues for all to consider and potential action points that will lead towards a more inclusive research landscape. The Community Voices Comments are opinion articles that give an opportunity to the authors to express their personal views and /or share action plans and three are published alongside this editorial. One by Gabriela Auge et al. outlines the importance of building diverse networks of mentorships to foster diversity, equity, and inclusion and another by Jory Lerback et al. elaborates on how structural changes at the level of research institutions should be favored over solutions aimed at increasing resilience of individual researchers from minoritized backgrounds; a final article by Marie Bernard et al. present the NIH strategies to address gender discrimination with the goal of fostering innovation and creativity. We are sure that there are many more key points and initiatives that should be brought forward and we invite you to contact us with your ideas.

